# The Effects of Non-Invasive Radiofrequency Treatment and Hyperthermia on Malignant and Nonmalignant Cells

**DOI:** 10.3390/ijerph110909142

**Published:** 2014-09-03

**Authors:** Steven A. Curley, Flavio Palalon, Kelly E. Sanders, Nadezhda V. Koshkina

**Affiliations:** 1 Department of Surgical Oncology, Baylor College of Medicine, Houston, TX 77030, USA; E-Mails: steven.curley@bcm.edu (S.A.C.); flavio.palalon@gmail.com (F.P.); Kelley.e.sanders@rice.edu (K.E.S.); 2 Department of Mechanical Engineering and Materials Science, Rice University, Houston, TX 77030, USA

**Keywords:** electromagnetic waves, hyperthermia, cancer, autophagy, mitochondria, proliferation

## Abstract

Background: Exposure of biological subjects to electromagnetic fields with a high frequency is associated with temperature elevation. In our recent studies, we reported that non-invasive radiofrequency (RF) treatment at 13.56 MHz with the field ranging from 1 KeV to 20 KeV/m^2^ inhibits tumor progression in animals with abdominal tumor xenografts and enhances the anticancer effect of chemotherapy. The RF treatment was followed by temperature elevation in tumors to approximately 46 °C during 10 min of exposure. In contrast, the temperature of normal tissues remained within a normal range at approximately 37 °C. Whether all biological effects of RF treatment are limited to its hyperthermic property remains unclear. Here, we compared how RF and hyperthermia (HT) treatments change the proliferation rate, oxygen consumption and autophagy in malignant and nonmalignant cells. Methods: In the current study, cancer and nonmalignant cells of pancreatic origin were exposed to the RF field or to conventional HT at 46 °C, which was chosen based on our previous *in vivo* studies of the tumor-specific RF-induced hyperthermia. Results: Only RF treatment caused declines in cancer cell viability and proliferation. RF treatment also affected mitochondrial function in cancer cells more than HT treatment did and, unlike HT treatment, was followed by the elevation of autophagosomes in the cytoplasm of cancer cells. Importantly, the effects of RF treatment were negligible in nonmalignant cells. Conclusion: The obtained data indicate that the effects of RF treatment are specific to cancer cells and are not limited to its hyperthermic property.

## 1. Introduction

Among the methods for cancer treatment, only systemic chemotherapy, targeted therapy, immunotherapy and ionizing radiation therapy are non-invasive. Other methods, such as cryotherapy, where the use of extreme cold procedures allows for the destruction of local tumors, or hyperthermia (HT) and radiofrequency ablation, where temperatures higher than 40 °C are used for the same purpose, in most cases require some surgical manipulations, such as implantation of treatment probes inside the tumor, and have limited applications [1,2,3,4]. In fewer cases, when the tumor is located on the skin or just below the skin, cryotherapy or HT can be applied externally with no surgical intervention. Non-invasive whole-body HT can be used to treat metastatic disease. During this procedure, the body temperature may be raised up to approximately 42–43 °C; this temperature can be reached in humans during fever. This causes a significant systemic shock in patients and, according to reports from the U.S. National Institute of Health, may cause severe cardiovascular toxicity and blood clotting. 

We recently reported the feasibility and safety of a novel, non-invasive RF therapeutic approach based on the use of electromagnetic waves with 13.56 MHz frequency for the treatment of orthotopic or subcutaneous liver and pancreatic cancers in mice [5,6,7]. Measurements of the temperatures inside tumors and in normal tissues during RF exposure indicated that this treatment can induce tumor-specific HT [7]. The temperature in orthotopic tumors elevated gradually, reaching 42 ± 2 °C after 5 min of exposure with a maximum temperature of 46 ± 2 °C achieved after 10 min of exposure. Importantly, healthy organs remained at the range of normal temperature. Indeed, cancer cells are known to be more sensitive to elevated temperatures than normal cells are [8], and the ability of HT to enhance the anticancer effects of chemotherapy is well-known [9,10].

Whether all anticancer effects that we observed previously in our *in vivo* and *in vitro* studies of cancer cells after RF treatment [5,7,11] were mediated only by its hyperthermic property remains unclear. Some studies indicate that electromagnetic fields with a frequency of less than 300 GHz, corresponding to the range of radio waves and known to be non-heating, can also generate biological alterations and affect the growth of cancer cells and enhance the antiproliferative effect of chemotherapy [12,13,14,15,16]. Understanding the contribution of different components of RF treatment will be very important for further development of this novel, non-invasive therapeutic approach using RF fields. In the current project, we compared the effects of RF treatment with conventional HT treatment on cell proliferation, mitochondrial activity and autophagy in malignant and nonmalignant cells of pancreatic origin. Since, in our previous studies, we determined the highest temperature of 46 °C achieved in orthotopic tumors in mice after RF exposure [7], we selected this temperature for the conventional HT treatment.

## 2. Results

### 2.1. RF Treatment, But Not HT Treatment, Inhibited the Proliferation of Pancreatic Cancer Cells In Vitro

**Figure 1:**
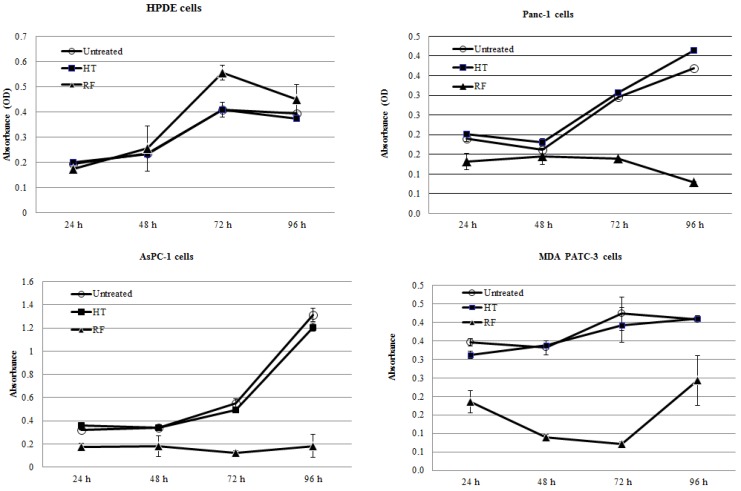
RF, not hyperthermia (HT), treatment inhibited proliferation of pancreatic cancer cells. HPDE, nonmalignant pancreatic ductal epithelial.

Three human pancreatic cancer cell lines (Panc-1, MDA PATC-3, AsPC-1) and nonmalignant pancreatic ductal epithelial (HPDE) cells were exposed to the RF field at 13.56 MHz for 5 min. Exposure of cancer cells to the RF treatment resulted in significant growth arrest when compared with untreated cells (*p* < 0.001, Figure 1). Two of the cancer cell lines, AsPC-1 and Panc-1, were not able to restore their proliferation activity for four days after RF exposure. MDA PATC-3 cancer cells recovered and slowly resumed proliferation three days after RF treatment, as shown by an MTT assay. In contrast to cancer cells, nonmalignant HPDE cells showed higher proliferation activity after RF exposure when compared with untreated HPDE cells. Of note, the bulk temperature of the medium containing the cells during RF exposure did not exceed 40 ± 2 °C. 

Pancreatic cancer cells, Panc-1, AsPC-1, MDAPATC-3, and normal pancreatic HPDE cells were exposed to HT or RF treatment for 5 min. Cell proliferation was determined by the MTT assay.

Exposure of cancer cells to conventional HT at 46 °C for 5 min was not significantly cytotoxic for pancreatic cancer cells and for normal HPDE cells (*p* > 0.1). 

**Figure 2:**
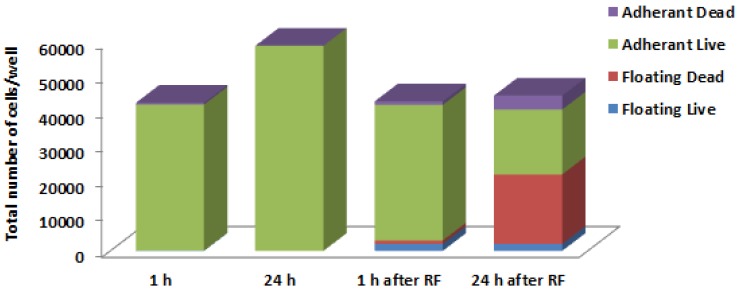
Distribution of adherent and detached Panc-1 cells after RF exposure and their viability.

More detailed studies of the cell’s behavior following RF treatment under the microscope showed that some cancer cells exposed to the RF field detached from the plate. The largest percent of detached cells, more than 50%, was observed in Panc-1 cells 24 h after the end of RF treatment. We calculated the viability of floating and adhered populations of these cells at 1 h and 24 h after RF exposure using the trypan blue assay. Analysis of untreated Panc-1 cells revealed the dominant presence of viable adherent cells, whose population increased after 24 h, as expected (Figure 2). In contrast, the percent of detached Panc-1 cells after RF exposure increased from approximately 5%–10% to over 50 % after 1 h and 24 h, respectively. The majority of floating cells 1 h after RF treatment remained viable, whereas after 24 h, 90% of them were dead. The number of viable adherent cells declined with time in RF-treated Panc-1 cells.

Panc-1 cells were exposed for 5 min to the RF field. Floating and adhered cells were harvested and counted with trypan blue dye for viability at the indicated time points.

### 2.2. RF Treatment Decreases Oxygen Consumption Rates (OCR) in Cancer Cells More than HT Treatment

**Figure 3:**
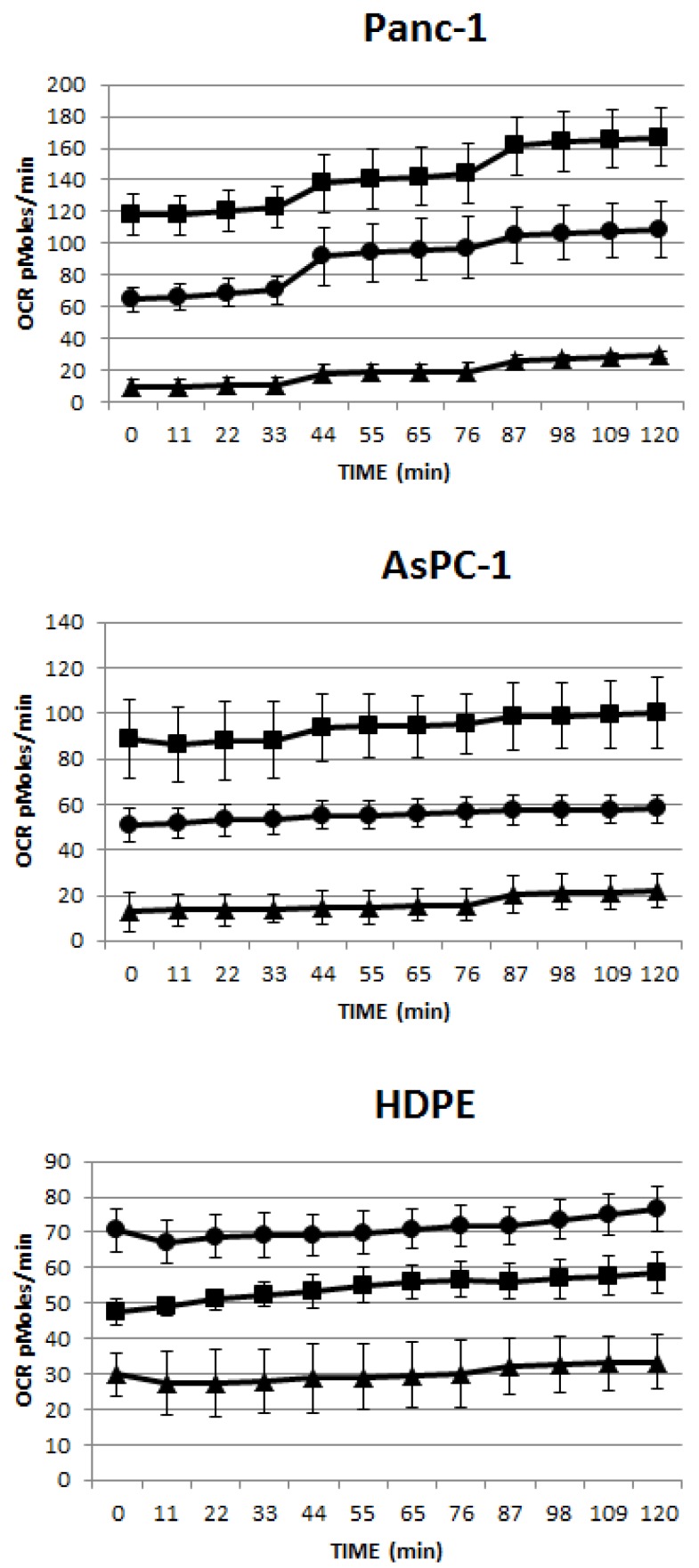
RF treatment decreased the oxygen consumption rate (OCR) more in cancer than normal cells and was superior compared to HT

The MTT assay we used to determine the cytostatic effect of RF treatment on cancer cells in the previous experiment is based on the alteration of redox potential inside cells, which is known to reflect mitochondrial activity [17]. Recently, we demonstrated the ability of RF treatment to directly impair the function of mitochondria in pancreatic cancer cells, altering mitochondrial membrane potential, impairing mitochondrial respiration and the elevation of reactive oxygen species production [18]. To compare the effects of RF and HT treatments on mitochondrial respiration in cells, we measured oxygen consumption rate (OCR) levels in malignant cells and nonmalignant cells after 5 min of exposure to the RF field or to HT. Pancreatic cancer cells Panc-1 and AsPC-1 cells were more susceptible to RF treatment than to HT treatment. RF treatment of cancer cells resulted in almost a 90% reduction of OCR levels compared to those in untreated cancer cells (*p* < 0.0001, Figure 3), whereas in HT-treated cancer cells, OCR cells were reduced by approximately 50% (*p* < 0.001).

Nonmalignant HPDE cells were less sensitive to both RT and HT treatments. Their OCR levels decreased only 50% after RF exposure when compared with untreated HPDE cells (*p* < 0.001). Interestingly, HT treatment caused the elevation of OCR levels in HPDE cells (*p* < 0.001).

Cancer cells Panc-1 and AsPC-1 and normal HPDE cells were exposed to RF (black triangles) or HT (black circles) treatment for 5 min, and OCR was measured 24 h after treatment. Untreated cells (black squares) were used as the control.

### 2.3. RF Treatment, But Not HT Treatment, Significantly Increased the Number of Autophagosomes in Cancer Cells

**Figure 4:**
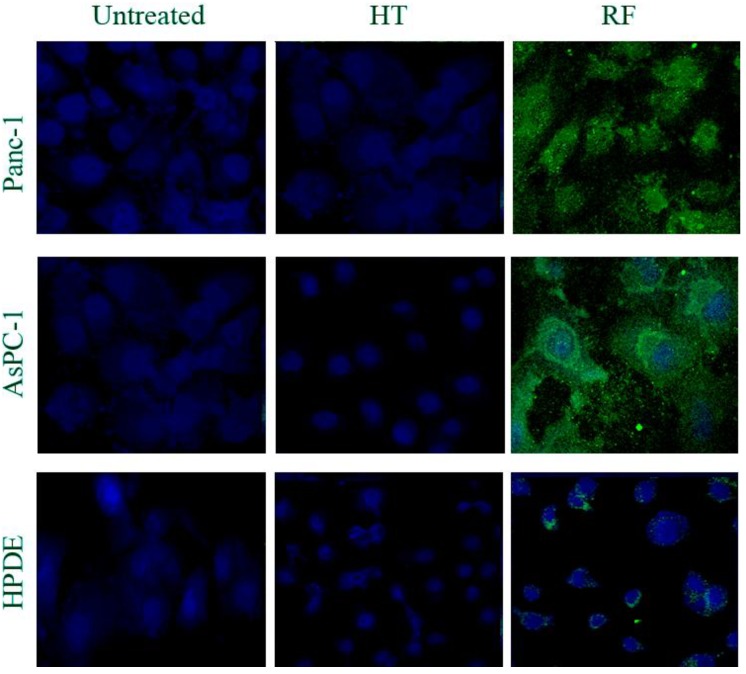
RF, not HT treatment, increases the number of LC3B-positive autophagosomes in malignant, but not normal, cells.

Our recent studies with RF treatment showed its ability to induce autophagy, not apoptosis, in pancreatic cancer cells [5]. Using multiple molecular approaches, we were able to demonstrate RF-stimulated conversion of the microtubule-associated protein 1B-light chain 3 molecule (LC3B I) to its lapidated LC3B-II form, specific for the autophagosome membrane. This was followed by elevation of GFP-LC3-positive autophagosomes in the cytoplasm of cancer cells transduced with lentiviral GFP-LC3 plasmid. Interestingly, we did not observe autophagy induction in nonmalignant cells after RF exposure. Here, we used anti-LC3B II antibody and the fluorescent immunocytochemical approach to assess the level of autophagosomes in cells after RF and HT treatments. As expected, 5 min of RF exposure caused significant elevation of green fluorescent LC3B II puncta in cancer cells, but not in nonmalignant HPDE cells (Figure 4). In contrast, after HT treatment, fluorescent levels of LC3B puncta were negligible in all cancer and nonmalignant cells and did not differ from the levels in the untreated cells.

Panc-1 and AsPC-1 cells were exposed to RF or HT treatment for 5 min and fixed 24 h after that. Cells were stained with primary anti-human LC3B antibody followed by FITC-labeled secondary antibody to detect autophagosomes (green staining). DAPI staining was used to identify nucleus (blue staining).

## 3. Discussion

Results obtained in the current studies demonstrated that the biological effects of RF treatment differ from those caused by conventional HT. Though some studies indicate the ability of HT to inhibit the proliferation rate of malignant cells, methodic analysis of HT’s effect on cancer cells showed that the cells’ growth rate represented a curve with exponential reduction in clonogenic survival as a function of time at the temperature range of 43–47 °C [19,20]. In other words, short exposure of cancer cells to high temperatures may induce stress in cells without significant inhibition of their proliferation rates, whereas long exposure even to the moderate temperatures may be toxic and lead to cell growth arrest. Therefore, the fact that in our experiments, the 5-min HT treatment had no effect on either cancer or nonmalignant cell viability and proliferation is not surprising, because in studies by other investigators, the significant decline in cancer cell growth could be achieved only when cells were exposed to such temperatures for 30–60 min [21]. In contrast to HT, exposure of cancer cells to the RF field for 5 min was sufficient to arrest or inhibit the proliferation of all tested pancreatic cancer cells without affecting nonmalignant cells. 

We were concerned about the ability of RF treatment to induce the detachment of some cancer cells from the surface of the culture dish. Detached cells may turn into metastatic cells and initiate tumor formation in the distant sites of the body. Careful examination of floating Panc-1 cells 24 h after RF exposure revealed that the majority of detached cells were dead. 

RF treatment substantially decreased mitochondrial activity in cancer cells (as shown by a 90% reduction of OCR levels), almost twice more than HT treatment did. The damaging effect of RF on mitochondria in cancer cells, at least in part, can explain the ability of RF to arrest cancer cell proliferation. The effect of RF on OCR levels in normal HPDE cells was similar to the effect of HT in cancer cells, causing a 50% reduction of OCR levels. Since we did not observe the inhibition of cell growth in these last cases, we concluded that 50% of OCR decline was not sufficient to cause significant changes in cellular proliferation mechanisms. The effect of HT on mitochondria in normal cells was quite opposite to the one noticed for the RF treatment and was followed by the elevation of OCR. These observations indicate that RF treatment and HT have a different action on the “breathing” function of cellular mitochondria and warrant further detailed study of this phenomenon.

We previously showed that RF treatment induced autophagy, but not apoptosis, in cancer cells of pancreatic origin [5]. In the present study, when we compared the levels of LC3B-positive autophagosomes in cancer cells and in nonmalignant cells after RF treatment and HT treatment, we observed that conventional HT did not increase levels of LC3B expression in all tested cells, showing its negligible effect on autophagy. In contrast, RF treatment led to much higher levels of LC3B puncta, indicative of autophagosomes, in cancer cells than in nonmalignant cells, thus demonstrating the ability of the RF field to stimulate autophagy in cancer cells.

Electromagnetic fields of ionizing radiation can induce non-thermal biological effects, but little is known about the specific effects of non-ionizing RF radiation in humans [22,23]. The currents produced by RF fields are known to interact with charged or polarized particles; since most biological molecules are charged, and since cell cytoplasm contains many ions, these molecules may respond to RF exposure and generate electrical fields and currents inside living cells. This type of interaction has been shown in studies with the use of electromagnetic waves of low intensity and intermediate frequency that did not generate heat [24,25]. Few other reports demonstrated the ability of RF fields to cause spindle disorientation of tubulin molecules and change their structure [26,27,28]. However, the biological effect of RF fields is not limited to structural modifications of tubulin molecules. Marchionni *et al.* reported the ability of RF fields to cause changes in ion channels [29]. Our current study contributes to these findings by showing that non-ionizing RF fields may simultaneously affect several other mechanisms, such as the inhibition of mitochondrial function and stimulation of autophagy, both of which may contribute to the cytostatic effect of RF treatment on cancer cells. Moreover, RF-induced biological effects on cancer cells exceeded those induced by HT treatment and caused minimal changes in nonmalignant cells. 

## 4. Methods

### 4.1. Chemical Reagents and Cell Culture

Panc-1 and AsPC-1 human cancer cells were acquired from the ATCC (Manassas, VA, USA). The MDA PATC-3 human pancreatic cancer cell line and normal pancreatic HPDE cells were obtained from Dr. Craig Logsdon (The University of Texas MD Anderson Cancer Center) and maintained as described elsewhere [30]. Panc-1 and MDAPATC-3 cancer cells were maintained in DMEM media supplemented with 10% fetal calf serum (FCS). AsPC-1 cells were maintained in MEM media containing 10% of FCS. For each cell line, the short tandem repeat fingerprint was confirmed by the Cell Line Characterization Core Service at MD Anderson Cancer Center within 1 year of the time the experiments were conducted. All media and supplements were purchased from Gibco (Life Technologies, Grand Island, NY, USA). The cells were passaged approximately every 5–6 days before reaching confluency. Media were replaced every 3 days.

### 4.2. RF and HT Treatments

A Kanzius RF generator (Therm Med LLC, Erie, PA, USA) was used to expose cells to a non-invasive external RF field as described elsewhere [5,7]. Briefly, the generator operates at an adjustable output power (0–2 kW) with a fixed frequency of 13.56 MHz. The generator was connected to a high-Q coupling system with a transmitter and a reciprocal platform at the bottom. The two heads were positioned 3.5 inches apart. The coaxial end-fire circuit in the transmitter head produces a uniform RF electrical field up to 15 cm in diameter. The field generated is predominantly electrical, with a minimal magnetic component.

For RF treatment, cells were seeded in 12-well plates in 1 mL medium or in 3-cm culture dish in 2 mL medium and incubated for 16–24 h. After that, the medium was replaced to remove non-attached and dead cells, and plates were placed between the transmitter and reciprocal platform, as shown in Figure 1, and exposed to the RF field for 5 min using parameters previously shown to be effective for killing cancer cells [5,7]. The temperature of the bulk medium over the cells was recorded digitally by the infrared thermal camera (FLIR SC-6000, FLIR SC-Systems, Inc., Boston, MA, USA).

For conventional HT exposure, cells were seeded as described above, and plates with cells were placed in a water bath preheated to 46 °C for 5 min. We selected 46 °C for our HT experiments, because that was the tumor-specific temperature achieved in orthotopic pancreatic and liver tumors in animal models during RF exposure. After treatment, cells were stored in a tissue culture incubator at 37 °C until the time of analysis.

### 4.3. Cell Proliferation and Viability Assays

Cell proliferation was measured using a colorimetric assay with the MTT reagent (3-(4,5-dimethylthiazol-2-yl)2,5-diphenyltetrazolium bromide), as described elsewhere [31]. Briefly, cells were seeded in 12-well plates at 10%–20% confluency and exposed to the RF field or HT, as described above. Untreated cells served as the control. After treatment, the plates were placed back into the tissue-culture incubator for 24, 48, 72 or 96 h. At the day of analysis, the MTT reagent at 0.05 mg/mL was added to the culture medium over the cells, and the cells were incubated for 2 h more. After that, the plates were centrifuged to spin down floating cells and include them into analysis; the medium was removed and replaced with dimethyl sulfoxide to solubilize the reduced MTT salt. The optical density of the samples was read at a wavelength of 570 nm on a spectrophotometer.

The number and viability of detached cells after RF treatment were determined in the separate experiment for which 50,000 Panc-1 cells were seeded into 3-cm culture dishes and incubated overnight. On the following day, cells were exposed to the RF field for 5 min. Detached cells were harvested and counted with trypan blue dye after 1 h and 24 h following the treatment. Adherent cells were trypsinized and also counted with trypan blue. Trypan blue dye was added to cells to determine the number of viable and necrotic cells in floating and adherent cell populations. Cells with intact cytoplasm membranes remained trypan blue negative; cells with damaged membranes were stained blue and were counted as dead. The cell count was performed on a hemocytometer.

### 4.4. OCR Measurement

Mitochondrial activity was evaluated by measuring the OCR in pmol/min on an XF Extracellular Flux Analyzer (Seahorse Bioscience, Inc., North Billerica, MA, USA), according to the manufacturer’s instructions. At 24 h after RF exposure, cells were reseeded onto an XF96 cell culture plate in 0.1 mL of XF assay medium at 60,000 cells/well for AsPC-1 and HDPE cells or 40,000 cells/well for Panc-1 cells. The plate was centrifuged at 1200 rpm for 5 min to settle all cells at the bottom of the plate. The sensor cartridge was calibrated using XF calibration medium overnight prior to the day of analysis. The XF Extracellular Flux Analyzer was programmed to take the OCR reading every 10 min continually for 2 h.

### 4.5. Fluorescence Immunocytochemistry Analysis of Autophagy

To stain autophagosomes, cells were seeded and treated with RF and HT, as described above. After treatment, cells were returned to the incubator for 24 h, fixed with 2% paraformaldehyde and stained with primary antibody against LC3B protein (Cell Signaling Technology, Inc., Danvers, MA, USA) followed by FITC-conjugated secondary antibody. Nuclei were stained with DAPI. Cells were imaged under the inverted fluorescent microscope, Olympus IX81.

### 4.6. Statistical Methods

Each experiment was repeated at least three times. Results from experiments are presented as means with standard deviations from the mean. GraphPad Instat 3 software (GraphPad Software Inc., La Jolla, CA) was used for the evaluation of the distribution assumption of analysis and the validation of the test type for statistical analysis. All results showed a normal pattern of value distribution and were analyzed by the two-sided Student’s *t*-test; *p* < 0.05 was considered statistically significant.

## 5. Conclusions

The results obtained in the current study, along with our previous reports, indicate the ability of RF treatment to provide a tumor-specific cytotoxic effect by inhibiting the proliferation and mitochondrial activity of tumor cells and the stimulation of autophagy. These effects exceed the hyperthermic property of the RF field. All of this assures further investigation of the biological effects of RF treatment to stimulate the development of novel, non-invasive approaches for cancer treatment using electromagnetic fields.

## References

[1] Ding J., Jing X., Liu J., Wanf Y., Wang F., Wang Y., Du Z. (2013). Complications of thermal ablation of hepatic tumours: Comparison of radiofrequency and microwave ablative techniques. Clin. Radiol..

[2] Niu L., Xu K., Mu F. (2012). Cryosurgery for lung cancer. J. Thorac. Dis..

[3] Zhou G., Chiu D, Qin D., Niu L., Cai J., He L., Huang W., Xu K. (2012). The efficacy evaluation of cryosurgery in pancreatic cancer patients with the expression of CD44v6, integrin-beta1, CA199, and CEA. Mol. Biotechnol..

[4] Owusu-Agyemang P., Arunkumar R., Green H., Hurst D., Landoski K., Hayes-Jordan A. (2012). Anesthetic management and renal functional in pediatric patients undergoing cytoreductive surgery with continous hyperthermic intraperitoneal chemotherapy (HIPEC) with cisplatin. Ann. Surg. Oncol..

[5] Koshkina N.V., Briggs K., Palalon F., Curley S.A. (2014). Autophagy and enhanced chemosensitivity in experimental pancreatic cancers induced by noninvasive radiofrequency field treatment. Cancer.

[6] Glazer E.S., Zhu C., Massey K.L., Thompson C.S., Kaluarachchi W.D., Hamir A.N., Curley S.A. (2010). Noninvasive radiofrequency field destruction of pancreatic adenocarcinoma xenografts treated with targeted gold nanoparticles. Clin. Cancer Res.: An Offic. J. Am. Assoc. Cancer Res..

[7] Raoof M., Cisneros B.T., Corr S.J., Palalon F., Curley S.A., Koshkina N.V. (2013). Tumor selective hyperthermia induced by short-wave capacitively-coupled RF electric-fields. PloS ONE.

[8] Gerweck L.E. (1985). Hyperthermia in cancer therapy: The biological basis and unresolved questions. Cancer Res..

[9] Hayes-Jordan A., Green H., Ludwig J., Anderson P. (2012). Toxicity of hyperthermic intraperitoneal chemotherapy (HIPEC) in pediatric patients with sarcomatosis/carcinomatosis: Early experience and phase 1 results. Pediatr. Blood Cancer.

[10] Di Miceli D., Alfieri S., Caprino P., Menghi R., Quero G., Cina C., Ridolfini M.P., Doglietto G.B. (2012). Complications related to hyperthermia during hypertermic intraoperative intraperitoneal chemiotherapy (HIPEC) treatment. Do they exist?. Eur. Rev. Med. Pharmacol. Sci..

[11] Glazer E.S., Curley S.A. (2010). Radiofrequency field-induced thermal cytotoxicity in cancer cells treated with fluorescent nanoparticles. Cancer.

[12] Zimmerman J.W., Pennison M.J., Brezovich I., Yi N., Yang C.T., Ramaker R., Absher D., Myers R.M., Kuster N., Costa F.P. (2012). Cancer cell proliferation is inhibited by specific modulation frequencies. Br. J. Cancer.

[13] Kirson E.D., Dbalý V., Tovaryš F., Vymazal J., Soustiel J.F., Itzhaki A., Mordechovich D., Steinberg-Shapira S., Gurvich Z., Schneiderman R. (2007). Alternating electric fields arrest cell proliferation in animal tumor models and human brain tumors. Proc. Natl. Acad. Sci. USA.

[14] Sowers A.E. (1984). Characterization of electric field-induced fusion in erythrocyte ghost membranes. J. Cell Biol..

[15] Trosic I., Pavicic I., Marjanovic A.M., Busljeta I. (2012). Non-thermal biomarkers of exposure to radiofrequency/microwave radiation. Arh. Hig. Rada Toksikol..

[16] Andocs G., Renner H., Balogh L., Fonyad L., Jakab C., Szasz A. (2009). Strong synergy of heat and modulated electromagnetic field in tumor cell killing. Strahlenther. und Onkol..

[17] Indran I.R., Hande M.P., Pervaiz S. (2010). Tumor cell redox state and mitochondria at the center of the non-canonical activity of telomerase reverse transcriptase. Mol. Asp. Med..

[18] Curley S.A., Palalon F., Lu X., Koshkina N.V. (2014). Noninvasive radiofrequency treatment effect on mitochondria in pancreatic cancer cells. Cancer.

[19] Raaphorst G.P., Freeman M.L., Dewey W.C. (1979). Radiosensitivity and recovery from radiation damage in cultured CHO cells exposed to hyperthermia at 42.5 or 45.5 °C. Radiat. Res..

[20] Dewey W.C., Hopwood L.E., Sapareto S.A., Gerweck L.E. (1977). Cellular responses to combinations of hyperthermia and radiation. Radiology.

[21] Roti Roti J.L. (2008). Cellular responses to hyperthermia (40–46 °C): Cell killing and molecular events. Int. J. Hyperth..

[22] Jauchem J.R. (2008). Effects of low-level radio-frequency (3 kHz to 300 GHz) energy on human cardiovascular, reproductive, immune, and other systems: A review of the recent literature. Int. J. Hyg. Environ. Health.

[23] Szmigielski S. (2013). Reaction of the immune system to low-level RF/MW exposures. Sci. Total Environ..

[24] Kirson E.D., Gurvich Z., Schneiderman R., Dekel E., Itzhaki A., Wasserman Y., Schatzberger R., Palti Y. (2004). Disruption of cancer cell replication by alternating electric fields. Cancer Res..

[25] Kirson E.D., Giladi M., Gurvich Z., Itzhaki A., Mordechovich D., Schneiderman R.S., Wasserman Y., Ryffel B., Goldsher D., Palti Y. (2009). Alternating electric fields (TTFields) inhibit metastatic spread of solid tumors to the lungs. Clin. Exp. Metastasis.

[26] Taghi M., Gholamhosein R., Saeed R.Z. (2013). Effect of Radio Frequency Waves of Electromagnetic Field on the Tubulin. Recent Patents Endocr., Metab. Immune Drug Discov..

[27] Taghi M., Gholamhosein R., Saeed R.Z. (2012). Effect of electromagnetic field on the polymerization of microtubules extracted from rat brain. Recent Patents Endocr., Metab. Immune Drug Discov..

[28] Zimmerman J.W., Pennison M.J., Brezovich I., Yi N., Yang C.T., Ramaker R., Absher D., Myers R.M., Kuster N., Costa F.P. (2012). Cancer cell proliferation is inhibited by specific modulation frequencies. Br. J. Cancer.

[29] Marchionni I., Paffi A., Pellegrino M., Liberti M., Apollonio F., Abeti R., Fontana F., D’lnzeo G., Mazzanti M. (2006). Comparison between low-level 50 Hz and 900 MHz electromagnetic stimulation on single channel ionic currents and on firing frequency in dorsal root ganglion isolated neurons. Biochim. Biophys. Acta.

[30] Li M., Zhai Q., Bharadwaj U., Wang H., Li F., Fisher W.E., Chen C., Yao Q. (2006). Cyclophilin A is overexpressed in human pancreatic cancer cells and stimulates cell proliferation through CD147. Cancer.

[31] Koshkina N.V., Kleinerman E.S. (2005). Aerosol gemcitabine inhibits the growth of primary osteosarcoma and osteosarcoma lung metastases. Int. J. Cancer.

